# A taxonomic study on *semifumata* species-group of *Fissocantharis* Pic, with description of six new species from China and Myanmar (Coleoptera, Cantharidae)

**DOI:** 10.3897/zookeys.152.2070

**Published:** 2011-12-08

**Authors:** Yuxia Yang, Xingke Yang

**Affiliations:** 1 College of Life Sciences, Hebei University, Baoding 071002, Hebei Province, China; 2 Key Laboratory of Zoological Systematics and Evolution, Institute of Zoology, Chinese Academy of Sciences, Beijing 100101, China

**Keywords:** Coleoptera, Cantharidae, *Fissocantharis*, new species, China, Myanmar

## Abstract

The cantharid *Fissocantharis semifumata* species-groupis reviewed. *Fissocantharis semifumata* (Fairmaire, 1889) is redescribed and illustrated. The type series of *Fissocantharis fissa* (Wittmer, 1997) is shown to consist of 3 species and clarified, except the holotype, the two paratypes become invalid. *Fissocantharis grahami* (Wittmer, 1997) is attributed to this species group. Six new species are described and illustrated, *Fissocanthais yui* **sp. n.** (CHINA: Yunnan), *Fissocantharis semimetallica* **sp. n.** (CHINA: Yunnan; MYANMAR: Kachin), *Fissocantharis bicolorata* **sp. n.** (CHINA: Sichuan), *Fissocantharis maculiceps* **sp. n.** (CHINA: Gansu), *Fissocantharis bimaculata* **sp. n.** (CHINA: Sichuan) and *Fissocantharis flava* **sp. n.** (CHINA: Sichuan, Guizhou). The number of species in the *Fissocantharis semifumata* species-groupis increased from 4 to 11, and a key to all species is provided.

## Introduction

The genus *Fissocantharis* Pic, 1921 was synonymized with *Micropodabrus* Pic, 1920 by Wittmer (1997). It was reinstated to be valid and redefined by Yang et al. (2009). Until now, this genus has about 180 species (Yang and Yang 2009), which are widely distributed in the Oriental and East Palaearctic Regions (Kazantsev and Brancucci 2007).

The *semifumata* species-group of *Fissocantharis* was proposed by Švihla (2005) based on 4 species, *Fissocantharis semifumata* (Fairmaire, 1889), *Fissocantharis fissa* (Wittmer, 1997), *Fissocantharis semifumatoides* (Švihla, 2005) and *Fissocantharis fissiformis* (Švihla, 2005).

During our recent study on this species group, we discover that the aedeagus of *Fissocantharis semifumata* (Fairmaire) illustrated by Wittmer (1997) is different from that of the type specimen. Also, the type series of *Fissocantharis fissa* (Wittmer) is shown to consist of 3 species, except the holotype, the two male paratypes belong to different species respectively. Besides, *Fissocantharis grahami* (Wittmer, 1997) should be attributed to this species group because of its similarity to *Fissocantharis fissa* in both appearance and aedeagus. Except the above 5 species, 6 new species of this species group are described here under the names of *Fissocantharis yui* sp. n., *Fissocantharis semimetallica* sp. n., *Fissocantharis bicolorata* sp. n., *Fissocantharis maculiceps* sp. n., *Fissocantharis bimaculata* sp. n. and *Fissocantharis flava* sp. n. Now the *Fissocantharis semifumata* species-group has 11 species, which are all distributed in SW China, except one species spreads to NE Myanmar.

## Material and methods

The material of this study is deposited in the following collections:

HBUM	Hebei University Museum, Baoding, China
            

IZAS	Institute of Zoology, Chinese Academy of Sciences, Beijing, China
            

MNHN	Muséum National d'Histoire Naturelle, Paris, France
            

NHMB	Naturhistorisches Museum Basel, Switzerland
            

Labels of the type material are cited verbatim, some old names of localities are updated in square brackets [ ]. Names of localities of the additional material are written in standard English style, if in Chinese, annotated with transliterations in square brackets [ ]. The depositories of all material are noted at the end of their localities in round brackets ( ).

The aedeagi are detached from the body under a stereoscopic microscope and kept in 10% KOH solution for several minutes, then cleared in 75% alcohol and observed under a compound light microscope. Line illustrations are drawn with the aid of a camera lucida mounted on a Nikon SMZ 800 stereomicroscope. The scanning electronic micrographs are edited in CORELDRAW 12 and ADOBE PHOTOSHOP 8.0.1. The habitus photos are taken by Canon 450D digital camera with a Canon EF 100mm f/2.8 USM Macro Lens. The body length is measured from the anterior margin of clypeus to apex of elytron, and width is at the humeri of the conjoint elytra. Absolute measurements are used in millimetres (mm).

## Taxonomy

### Key to the species of *Fissocantharis semifumata* species-group (males)

**Table:** 

1	Elytra metallic blue or green or mixed with light yellow	2
–	Elytra yellow or light yellow, or mixed with black, never metallic	5
2	Elytra at most light yellow at bases of outer margins; pronotum with lateral margins slightly diverging posteriorly	3
–	Elytra at least light yellow at lateral margins and humeri; pronotum with lateral margins distinctly diverging posteriorly	4
3	Aedeagus: conjoint dorsal plate of parameres with lateral emarginations of apical margin shallow, protuberances between median and lateral emarginations slightly wide and nearly truncated at apices	*Fissocantharis yui* sp. n.
–	Aedeagus:conjoint dorsal plate of parameres with lateral emarginations of apical margin slightly deep, protuberances between median and lateral emarginations slightly narrow and rounded at apices	*Fissocantharis grahami* (Wittmer, 1997)
4	Femora entirely yellow; aedeagus: ventral process of each paramere slightly turned outwards at apex in lateral view, median lobe with a sclerotized projection in middle of dorsum	*Fissocantharis fissa* (Wittmer, 1997)
–	Femora black along apical two-thirds of upper sides; aedeagus: ventral process of each paramere distinctly turned outwards at apex in lateral view, median lobe without any sclerotized projection in dorsum	*Fissocantharis semimetallica* sp. n.
5	Aedeagus: ventral process of each paramere wide	6
–	Aedeagus: ventral process of each paramere narrow	9
6	Head with a black marking on vertex; aedeagus: conjoint dorsal plate of parameres with median emargination of apical margin as deep as lateral ones	*Fissocantharis maculiceps* sp. n.
–	Head without any black marking; aedeagus: conjoint dorsal plate of parameres with median emargination of apical margin deeper than lateral ones	7
7	Aedeagus: conjoint dorsal plate of parameres with median emargination of apical margin inverse-trapeziform, protuberances between median and lateral emarginations truncated at apices	*Fissocantharis flava* sp. n.
–	Aedeagus: conjoint dorsal plate of parameres with median emargination of apical margin not like above, protuberances between median and lateral emarginations rounded at apices	8
8	Elytra entirely light yellow, without black markings, lateral margins nearly parallel	*Fissocantharis semifumatoides* (Švihla, 2005)
–	Elytra yellow, each with a large rounded black marking at apex, lateral margins distinctly diverging posteriorly	*Fissocantharis bimaculata* sp. n.
9	Head behind eyes black; pronotum with black markings; aedeagus: conjoint dorsal plate of parameres with median emargination of apical margin shallower than lateral ones	*Fissocantharis fissiformis* (Švihla, 2005)
–	Head entirely yellow or reddish brown; pronotum without black markings; aedeagus: conjoint dorsal plate of parameres with the median emargination of apical margin deeper than lateral ones	10
10	Elytra with lateral margins diverging posteriorly; aedeagus: conjoint dorsal plate of parameres with median emargination of apical margin wide, protuberances between median and lateral emarginations narrow	*Fissocantharis semifumata* (Fairmaire, 1889)
–	Elytra with lateral margins nearly parallel; aedeagus: conjoint dorsal plate of parameres slightly with median emargination of apical margin slightly narrow, protuberances between median and lateral emarginations slightly wide	*Fissocantharis bicolorata* sp. n.

#### Fissocantharis semifumata

(Fairmaire, 1889)

http://species-id.net/wiki/Fissocantharis_semifumata

Figs 1–3 11–13

Podabrus semifumatus Fairmaire, 1889: 39.
Podabrus bicoloricornis Pic, 1926a: 356. – Wittmer, 1989: 219 (syn.).
Rhagonycha nigrosubapicalis Pic, 1926b: 5. – Wittmer, 1989: 219 (syn.).
Rhagonycha semifumata : Wittmer, 1989: 219.
Micropodabrus semifumatus : Wittmer, 1997: 312.
Fissocantharis semifumata : Yang et al. 2009: 49.

##### Type material examined.

Holotype ♂ of *Podabrus semifumatus* Fairmaire, 1889, “Moupin [Sichuan: Baoxing], 1870, A. David” (MNHN). Holotype ♂ of *Podabrus bicoloricornis* Pic, 1926, “Moupin [Sichuan: Baoxing], 1870, A. David” (MNHN). Holotype ♂ of *Rhagonycha nigrosubapicalis* Pic, 1926, “Szetschwan [Sichuan], Gunpanting [Songpan], Stöner” (MNHN).

##### Additional material examined.

2♂♂, CHINA, Sichuan, Mt. Emei, 500–1200m, 29.30°N, 103.20°E, 4–18.v.1989, leg. S.J. Kolibáč (NHMB); 1♂, same locality, 1000m, 4–20.v.1989, leg. Vit Kubáň (NHMB); 1♂, same locality, 600–1050m, 5–19.v.1989, lgt. Lad. Bocák (NHMB); 1♂, 1♀, Sichuan, Mt. Emei, 580–960m, 21.vi.1955, leg. Xingchi Yang [transliterated from Chinese label, the followings as the same] (IZAS); 1♀, same locality, 580–1150m, 27.vi.1955, leg. Zhonglin Ge (IZAS); 1♀, same locality, 1800–2100m, 24.vi.1955, leg. Zhonglin Ge (IZAS); 1♀, same locality, 2100–3100m, 25.vi.1955, leg. Le Wu (IZAS); 1♂, Sichuan, Chudian, 1783m, 23.vi.1957, leg. Fuxing Zhu (IZAS).

##### Distribution.

China (Gansu, Sichuan).

##### Redescription.

**Figures 1–4.**
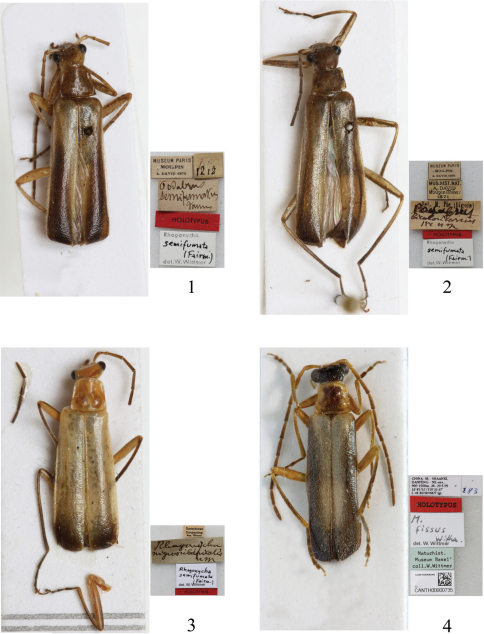
Male habitus, dorsal view **1–3** *Fissocantharis semifumata* (Fairmaire, 1889) **1** Holotype of *Podabrus semifumatus* Fairmaire, 1889 **2** Holotype of *Podabrus bicoloricornis* Pic, 1926 **3** Holotype of *Rhagonycha nigrosubapicalis* Pic, 1926 **4** Holotype of *Micropodabrus fissus* Wittmer, 1997

**Male**(Figs 1–3). Head yellow, apices of mandibles, terminal labial and maxillary palpomeres dark brown, antennae black, antennomeres I yellow, slightly darkened at apices, prothorax and scutellum yellow, elytra mostly black, light yellow at bases and lateral margins, of which inner margins slightly wider on anterior than posterior part and distinctly wider than outer margins, legs yellow, femora darkened at apices, tibiae black along upper sides, tarsi black, meso- and metasterna and abdomen black, last 2 abdominal ventrites yellow.

Head subquadrate, evenly narrowed behind eyes, densely and finely punctate, eyes moderately protruding, breadth across eyes slightly wider than anterior margin of pronotum, terminal maxillary palpomeres long-triangular, widest near apices, antennae filiform and simple, extending to apical one-third of elytra, antennomeres II slightly widened apically, about 1.5 times as long as wide at apices, III about twice as long as II, V longest, XI slightly longer than X.

Pronotum subquadrate, slightly wider than long, widest at base, anterior margin arcuate, lateral margins diverging posteriorly, posterior margin almost straight, anterior angles rounded, posterior angles nearly vertical, disc densely and finely punctate as that on head, distinctly convex on posterolateral parts.

Elytra about 4 times longer than pronotum, 3 times longer than humeral width, lateral margins distinctly diverging posteriorly, disc slightly sparsely and largely punctate than that on pronotum.

Legs: all tarsal claws bifid, with lower claws slightly shorter than upper ones.

Aedeagus (Figs 11–13): conjoint dorsal plate of parameres with median emargination of apical margin wide and distinctly deeper than lateral ones, protuberances between median and lateral emarginations narrow and almost half length of ventral process of each paramere in dorsal view; ventral process of each paramere narrow, slightly turned outwards at apex in lateral view; median lobe without any sclerotized projection in dorsum.

**Female.** Body larger, eyes less protruding and antennae shorter than that of males, pronotum distinctly wider than long, disc slightly convex, tarsal claws with lower claws distinctly shorter than upper ones.

##### Variation within species.

Sometimes elytra entirely light yellow, or slightly darkened at apices, legs with tibiae entirely black. Body length: 8.0–11.0 mm; width: 1.5–2.5 mm.

##### Remarks.

In this study, the holotype male of this species is examined, however, its male genitalia is different from the illustration provided by Wittmer (1997) who based on the specimen located in “Chasseurs Thibetains, Ta-Tsien-Lu, 1896” (NHMB). After a careful examination, the latter is shown to be a new species described below, *Fissocantharis bicolorata* sp. n. In this case, it is necessary to redescribe and illustrate this species here.

#### Fissocantharis fissa

(Wittmer, 1997)

http://species-id.net/wiki/Fissocantharis_fissa

Fig. 4

Micropodabrus fissus Wittmer, 1997: 313, Abb. 183.
Fissocantharis fissa : Yang et al. 2009: 49.

##### Type material examined.

Holotype ♂, “CHINA SE, Shaanxi, Danfeng NE env., 900–1500m, 33°45'N, 110°37'E33°52'N, 110°22'E, 28–29.v.1995, lgt. L.R. Businský" (NHMB).

##### Additional material examined.

1♂, CHINA, Hubei, Xingshan, Longmenhe, 1350m, 18.vi.1993, leg. Jian Yao; 1♂, same locality, 1280m, 23.vi.1993, leg. Wenzhu Li; 1♂, same locality, 1400m, 16.vi.1993, leg. Runzhi Huang; 1♀, same locality, 1260m, 14.vi.1993, leg. Hongxing Li; 1♀, same locality, 1280m, 14.vi.1993, leg. Jian Yao; 1♀, same locality, 1350m, 16.vi.1993, leg. Jian Yao; 1♀, same locality, 1350m, 14.vii.1993, leg. Baowen Sun [all transliterated from Chinese labels] (all in IZAS).

##### Distribution.

China (Shaanxi, Hubei).

##### Supplementary description.

**Male** (Fig. 4).Elytra metallic blue, light yellowatbases and lateral margins, of which inner margins slightly wider on anterior than posterior part and distinctly wider than outer margins.Aedeagus: conjoint dorsal plate of parameres with median emargination of apical margin wide and deeper than lateral ones, protuberances between median and lateral emarginations narrowed apically and rounded at apices, about one-fourth length of ventral process of each paramere in dorsal view; ventral process of each paramere slender; median lobe presenting with a sclerotized projection in middle of dorsum, which tapered apically and bent dorsally at apex.

**Female.** Body larger, eyes less protruding, antennae shorter and narrower than that of males, pronotum with disc slightly convex, elytra metallic blue, light yellow at bases of outer margins, all tarsal claws each with a triangular appendiculate.

Body length: 7.0–9.0 mm; width: 1.5–2.3 mm.

##### Remarks.

In the original manuscript by Wittmer (1997), the elytra of the type male was described as being partly black, which actually is metallic blue based on the examination of a series of additional specimens at our disposal. Also, it is the first time to describe the female for this species here.

Besides, the type series of this species is shown to be plural and composed of 3 species. Except the holotype, one male paratype labeled with “CHINA NW, Sichuan, Min Shan, 2500–4500m, 33°10'N, 103°50'E, 14–16.vii.1990, leg. Jiří Kolibáč” actually belongs to *Fissocantharis fissiformis* (Švihla, 2005), the other one “CHINA, Sichuan pr., Kangding distr., Hailougou Glacier Park, 21–24.vii.1992, lgt. R. Dunda” is a new species described here, *Fissocantharis bicolorata* sp. n. Thus, the two paratypes of this species designated by Wittmer (1997) become invalid. In this case, this species is excluded from Sichuan province at the moment.

#### Fissocantharis yui sp. n.

urn:lsid:zoobank.org:act:57C148E2-C9E5-476C-A8EA-81AC65B20EC0

http://species-id.net/wiki/Fissocantharis_yui

Figs 5 14–16 

##### Type material.

Holotype ♂, CHINA, Yunnan, Lanping, 13.vi.2010, leg. Guoyue Yu [transliterated from Chinese label] (IZAS). Paratypes: 2♂♂, same data to the holotype (IZAS).

##### Distribution.

China (Yunnan).

##### Diagnosis.

This species is similar to *Fissocantharis grahami* (Wittmer, 1997), but distinguishable by the aedeagus: conjoint dorsal plate of parameres with lateral emarginations of apical margin shallow, protuberances between median and lateral emarginations slightly wide and nearly truncated at apices.

##### Description.

**Figures 5–10.**
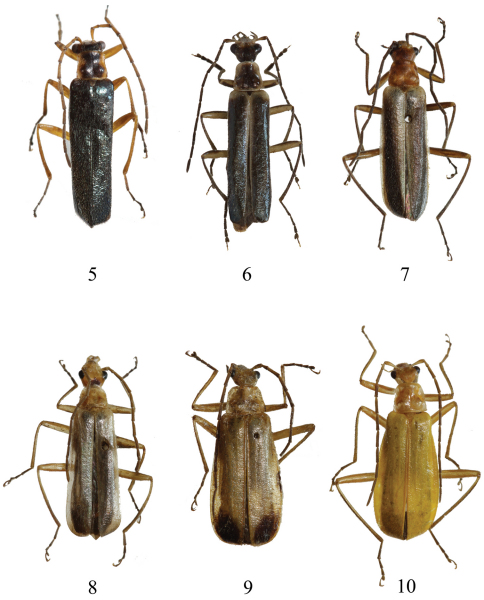
Male habitus, dorsal view **5** *Fissocantharis yui* sp. n. **6** *Fissocantharis semimetallica* sp. n. **7** *Fissocantharis bicolorata* sp. n. **8** *Fissocantharis maculiceps* sp. n. **9** *Fissocantharis bimaculata* sp. n. **10** *Fissocantharis flava* sp. n.

**Male** (Fig. 5). Head yellow, dorsum behind eyes black, apices of mandibles brown, terminal labial and maxillary palpomeres black, antennae black, antennomeres I–V yellow, darkened at apices, pronotum yellow, with a large black marking in middle, which extending from anterior to posterior margin and wider on posterior than anterior part, scutellum yellow, elytra metallic blue, light yellow at bases of outer margins, legs yellow, femora and tibiae darkened at apices, tarsi black, ventral parts of thorax and abdomen black, last 3 abdominal ventrites yellow.

Head subquadrate, evenly narrowed behind eyes, slightly depressed on vertex, dorsum densely and finely punctate, eyes slightly protruding, breadth across eyes wider than anterior margin of pronotum, terminal maxillary palpomeres slender, slightly widened near apices, antennae filiform and simple, extending to middle of elytra, antennomeres II about twice as long as wide, III one-third longer than II, XI slightly longer than X.

Pronotum subquadrate, almost as long as wide, widest at base, anterior margin arcuate, lateral margins slightly diverging posteriorly, posterior margin almost straight, anterior angles rounded, posterior angles nearly vertical, disc densely and finely punctate as that on head, convex on posterolateral parts.

Elytra about 5 times longer than pronotum, 4 times longer than humeral width, lateral margins parallel, disc densely and slightly largely punctate than that on pronotum.

Legs: all tarsal claws bifid, with lower claws slightly shorter than upper ones.

Aedeagus (Figs 14–16): conjoint dorsal plate of parameres with median emargination of apical margin slightly wide and distinctly deeper than lateral ones, protuberances between median and lateral emarginations slightly wide and nearly truncated at apices, about one-fourth length of ventral process of each paramere in dorsal view; ventral process of each paramere slender, slightly turned outwards in lateral view; median lobe presenting with a sclerotized lingulate projection in middle of dorsum.

**Female.** Unknown.

##### Type series variation.

Sometimes head black, slightly brown at clypeus, antennae entirely black, pronotum black, slightly brown at anterior and lateral margins, scutellum black, legs mostly dark brown. Body length: 6.0–7.0 mm; width: 1.1–1.3 mm.

##### Etymology.

Patronymic, dedicated to its collector, Dr. Guoyue Yu (Beijing, China).

#### Fissocantharis semimetallica sp. n.

urn:lsid:zoobank.org:act:B47A68A1-3F09-4879-B0D2-DCC04EB351AB

http://species-id.net/wiki/Fissocantharis_semimetallica

Figs 6 17–19 

##### Type material

Holotype ♂, MYANMAR, Kachin prov., Mt. Emaw Bum, road of Kanphant, 2358m, 26°09'N, 98°31'E, 28.v.2006, M. Langer (NHMB). Paratypes: 1♂, 2♀♀, same data as holotype (1♀ in NHMB; 1♂, 1♀ in IZAS); 1♂, CHINA, Yunnan Prov., Tengchong, Houqiao, Danzha, Zhaobitang, 2510m, 25.55627°N, 98.20941°E, 29.v.2006, H.B. Liang collector, California Academy & IOZ, Chinese Acad. Sci. (IZAS).

##### Distribution.

China (Yunnan), Myanmar (Kachin).

##### Diagnosis.

This new species is similar to *Fissocantharis fissa* (Wittmer, 1997), but can be distinguished by the femora black along apical two-thirds of upper sides, aedeagus: ventral process of each paramere distinctly turned outwards in lateral view, median lobe without any sclerotized projection in dorsum.

##### Description.

**Male** (Fig. 6).Head light yellow, dorsum behind eyes black, apices of mandibles dark brown, labial and maxillary palpomeres darkened, gula black, antennae black, pronotum black, light yellow at anterior and lateral margins, of which wider on anterior than posterior part, scutellum black, with very narrow light yellow lateral and apical margins, elytra metallic blue, light yellow at humeri and lateral margins, of which inner margins slightly wider than outer ones, legs black, coxae, trochanters and femora light yellow, femora black along apical two-thirds of upper sides, ventral parts of thorax and abdomen black, last abdominal ventrite light yellow.

Head subquadrate, evenly narrowed behind eyes, dorsum densely and finely punctate, eyes strongly protruding, breadth across eyes distinctly wider than anterior margin of pronotum, terminal maxillary palpomeres long-triangular, widest near apices, antennae filiform and simple, extending to apical one-third of elytra, antennomeres II slightly widened apically, about 3 times as long as wide at apices, III one-third longer than II, V longest, XI slightly shorter than X.

Pronotum subquadrate, almost as long as wide, widest at base, anterior margin arcuate, lateral margins distinctly diverging posteriorly, posterior margin almost straight, anterior angles rounded, posterior angles nearly vertical, disc densely and finely punctate as that on head, distinctly convex on posterolateral parts.

Elytra about 5 times longer than pronotum, 4 times longer than humeral width, lateral margins parallel, disc slightly sparsely and largely punctate than that on pronotum.

Legs: all tarsal claws bifid, with lower claws slightly shorter than upper ones.

Aedeagus (Figs 17–19): conjoint dorsal plate of parameres with median emargination of apical margin wide and distinctly deeper than lateral ones, protuberances between emarginations slightly narrow, about one-fourth length of ventral process of each paramere in dorsal view; ventral process of each paramere slender, distinctly turned outwards in lateral view; median lobe without any sclerotized projection in dorsum.

**Female.** Body larger, eyes less protruding, antennae shorter and narrower, pronotum wider than that of males, head mostly black, elytra with lateral margins diverging posteriorly, legs with coxae and femora black, light yellow at bases of ventral sides of femora, all tarsal claws each with a triangular appendiculate.

##### Type series variation.

Sometimes head and pronotum entirely light yellow, elytra light yellow almost at basal half part. Body length: 8.5–12.0 mm; width: 1.5–2.5 mm.

##### Etymology.

This new specific name is derived from Latin *semi* (half) and Greek *metall* (metallic), referring its elytra partly metallic blue.

##### Remarks.

One male paratype (CHINA, Yunnan) with left antennomeres VIII–XI, right protarsomeres II–V, right mesoleg, left mesotarsomeres II–V, right metatarsi and left metatarsomeres III–V and one female paratype with left antennomeres VII–XI, right VIII–XI and right metatarsus are missing.

**Figures 11–13.**
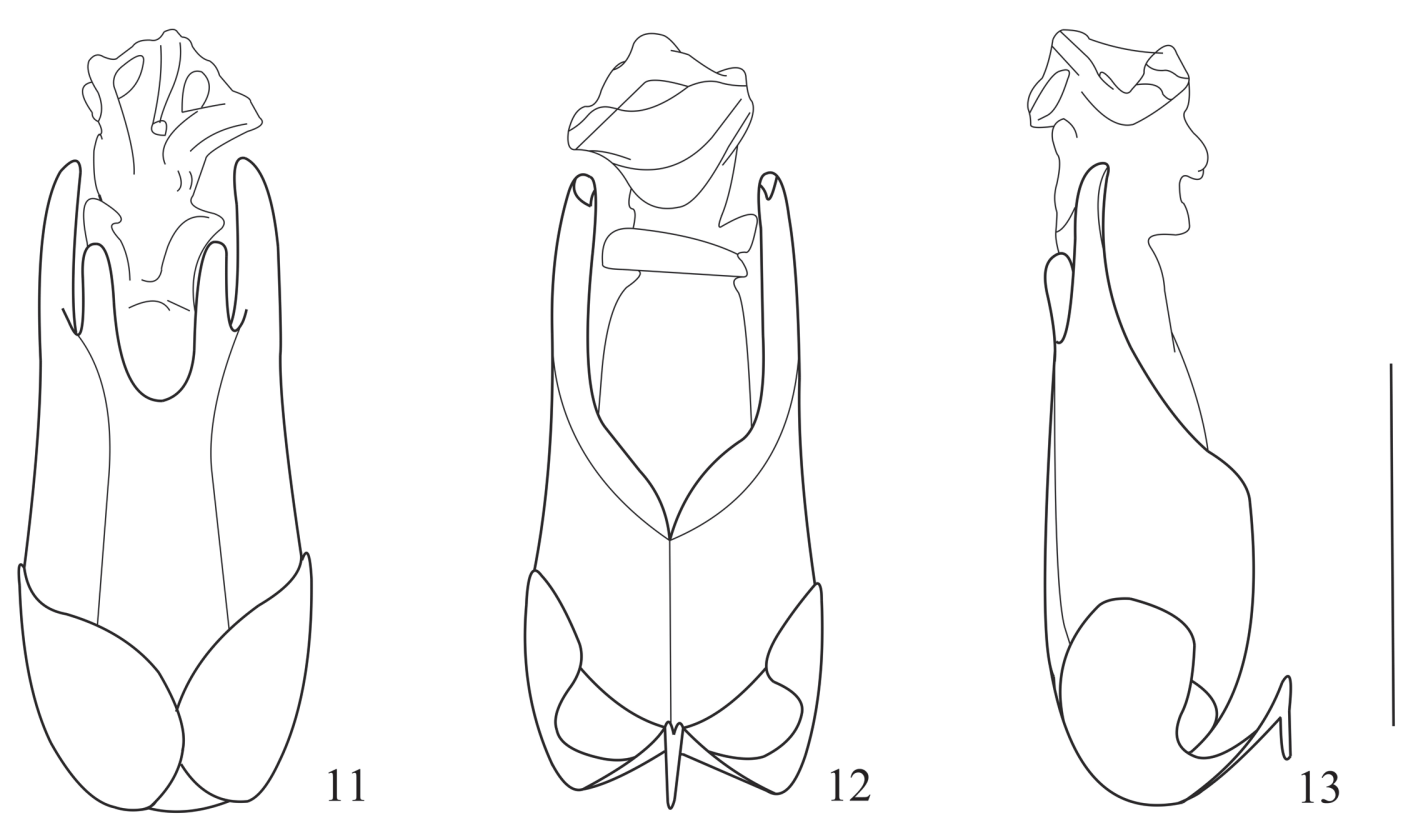
Aedeagus of *Fissocantharis semifumata* (Fairmaire, 1889) **11** dorsal view **12** ventral view **13** lateral view. Scale bar: 1 mm.

#### Fissocantharis bicolorata sp. n.

urn:lsid:zoobank.org:act:A1A55A22-BBA6-498D-AC18-34A5C42A4849

http://species-id.net/wiki/Fissocantharis_bicolorata

Figs 7 20–22

Micropodabrus semifumatus : Wittmer, 1997: 312, Abb. 184 [misidentification, nec. Fairmaire, 1889].

##### Type material.

Holotype ♂, CHINA, Sichuan, Kangding, Zheduoshanya, 4300m, 21.vi.1990, leg. Fusheng Huang [transliterated from Chinese label] (IZAS). Paratypes: 1♂, Sichuan pr., Kangding distr., Hailuogou Glacier Park, 21–24.vii.1992, lgt. R. Dunda (NHMB); 2♂♂, 1♀, Tat-sien-lu [Sichuan: Dajianlu], 1896 (NHMB); 1♂, Ta-tsien-Loû [Sichuan: Dajianlu], Chasseurs Thibetains, 1896 (NHMB); 2♂♂, Sichuan, Kangding, 2700m, 29.v.1983, leg. Xuezhong Zhang [transliterated from Chinese label, the followings as the same] (IZAS); 1♂, same locality, 2100m, leg. 22.vi.1983, leg. Shuyong Wang (IZAS); 2♂♂, same locality, 2500m, leg. 26.vi.1983, leg. Shuyong Wang (IZAS); 1♂, 1♀, same locality, 2600m, leg. 30.vi.1983, leg. Yuanqing Chen (IZAS); 1♀, same locality, 2300m, leg. 27.v.1983, leg. Yuanqing Chen (IZAS); 2♂♂, Sichuan, Ganzi, 3300m, 30.vi.1983, leg. Yuanqing Chen (IZAS); 3♀♀, Sichuan, Kangding, 3.vi.2004, leg. Yibin Ba & Aimin Shi (HBUM).

##### Distribution.

China (Sichuan).

##### Diagnosis.

This new species is similar to *Fissocantharis semifumata* (Fairmaire, 1889), but differs in the following characters: elytra with different coloration in both sexes, mixed black with light yellow in male, while entirely lightly yellow in female, lateral margins nearly parallel in male; aedeagus: conjoint dorsal plate of parameres with median emargination of apical margin slightly narrow, protuberances between median and lateral emarginations slightly wide.

##### Description.

**Male** (Fig. 7).Head reddish brown, clypeus light yellow, mouthparts dark brown, antennae black, pronotum and scutellum reddish brown, elytra black, light yellow at bases and lateral margins, of which inner margins wider than outer ones, legs black, femora light brown at inner sides, ventral parts of thorax and abdomen black, last 2 abdominal ventrites light yellow.

Head subquadrate, evenly narrowed behind eyes, dorsum densely and finely punctate, eyes moderately protruding, breadth across eyes slightly wider than anterior margin of pronotum, terminal maxillary palpomeres long-triangular, widest near apices, antennae filiform and simple, extending to apical one-third of elytra, antennomeres II about 1.5 times as long as wide at apices, III one-third longer than II, V longest, XI slightly shorter than X.

Pronotum subquadrate, slightly wider than long, widest at base, anterior margin arcuate, lateral margins diverging posteriorly, posterior margin almost straight, anterior angles rounded, posterior angles nearly vertical, disc densely and finely punctate as that on head, distinctly convex on posterolateral parts.

Elytra about 5 times longer than pronotum, 4 times longer than humeral width, lateral margins nearly parallel, disc slightly sparsely and largely punctate than that on pronotum.

Legs: all tarsal claws bifid, with upper claws almost as long as lower ones.

Aedeagus (Figs 20–22): conjoint dorsal plate of parameres with median emargination of apical margin slightly narrow and deeper than lateral ones, protuberances between median and lateral emarginations slightly wide and rounded at apices, about half length of ventral process of each paramere in dorsal view; ventral process of each paramere narrow, slightly turned outwards at apex in lateral view; median lobe without any sclerotized projection in dorsum.

**Female.** Body larger, eyes less protruding, antennae narrower and shorter than that of males, pronotum distinctly wider than long, elytra entirely light yellow, with lateral margins diverging posteriorly, tarsal claws with lower claws distinctly shorter than upper ones.

Body length: 8.0–11.0 mm; width: 1.5–2.5 mm.

##### Etymology.

This new specific name is derived from Latin *bi-* (two) and *color* (coloration), referring to it being sexually dimorphic in coloration of elytra.

**Figures 14–22.**
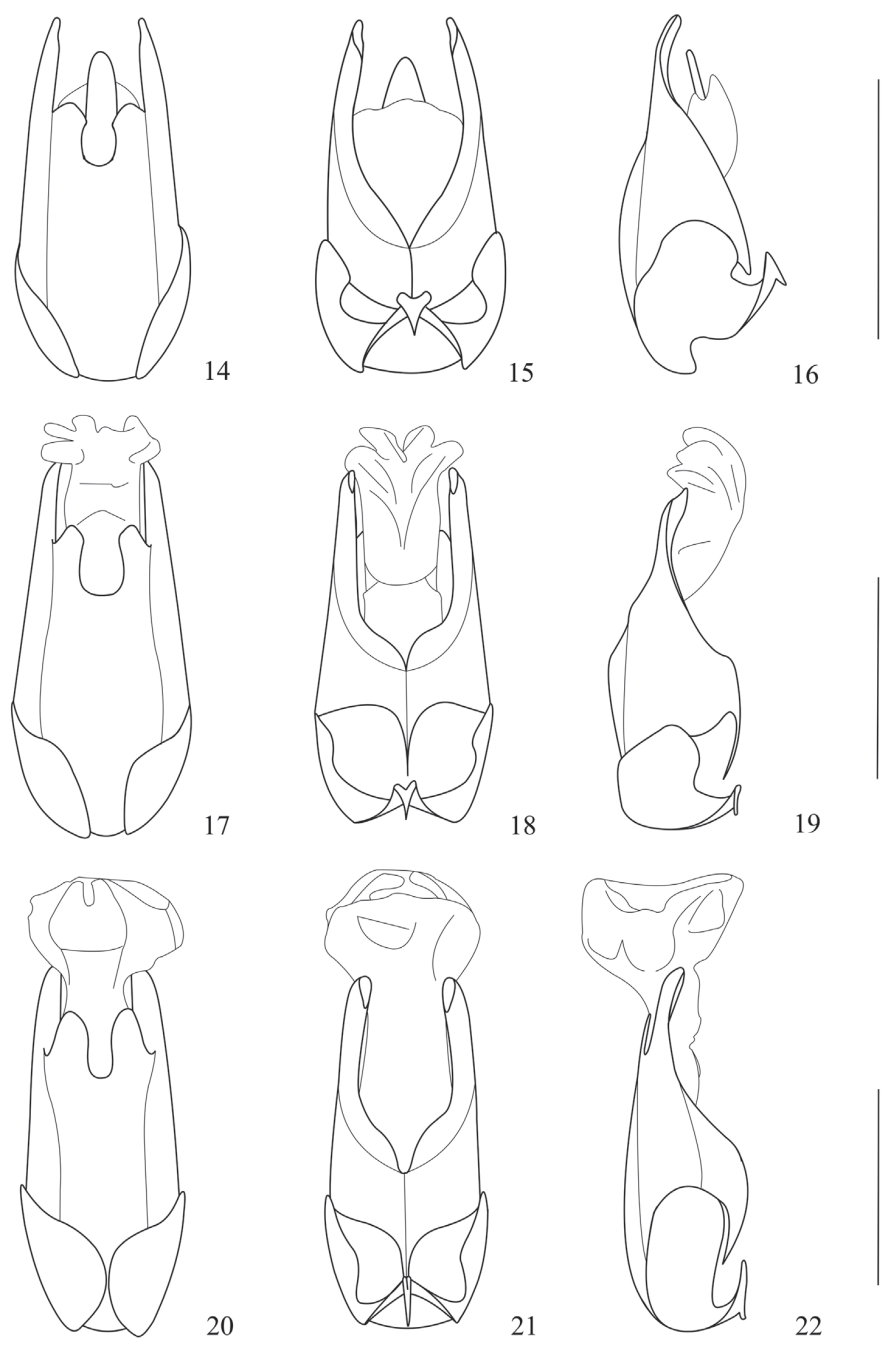
Aedeagi **14–16** *Fissocantharis yui* sp. n. **17–19** *Fissocantharis semimetallica* sp. n. **20–22** *Fissocantharis bicolorata* sp. n. **14, 17, 20** dorsal view **15, 18, 21** ventral view **16, 19, 22** lateral view. Scale bars: 1 mm.

#### Fissocantharis maculiceps sp. n.

urn:lsid:zoobank.org:act:64BAA203-6045-448D-9978-05EF2FB85AC2

http://species-id.net/wiki/Fissocantharis_maculiceps

Figs 8 23–25 

##### Type material.

Holotype ♂, CHINA, Gansu, Wenxian, Huangtuling, 2350m, 9.vii.2003, leg. Yibin Ba &Yang Yu (HBUM). Paratypes: 2♂♂, 2♀♀, same data to the holotype (1♂, 1♀ in HBUM; 1♂, 1♀ in NHMB); 1♂, Gansu, Wenxian, Qiujiaba, 2350–2650m, 30.vi.1998, leg. Xingke Yang (IZAS); 1♂, same locality, 2000–2100m, 1.vii.1998, leg. Shuyong Wang (IZAS); 1♀, same locality, 2200–2350m, 29.vi.1998, leg. Decheng Yuan (IZAS); 1♀, same locality, 2350–2650m, 30.vi.1998, leg. Decheng Yuan (IZAS); 2♀♀, same locality, 2350m, 28.vi.1998, leg. Jian Yao (IZAS); 1♀, same locality, 2350–2650m, 30.vi.1998, leg. Wenyi Zhou (IZAS); 3♀♀, Gansu, Zhouqu, Shatan Forestry, 2400m, 6.vii.1999, leg. Jian Yao (IZAS) [all transliterated from Chinese labels].

##### Distribution.

China (Gansu).

##### Diagnosis.

This new species is similar to *Fissocantharis fissiformis* (Švihla, 2005), but can be distinguished by the aedeagus: conjoint dorsal plate of parameres with median emargination of apical margin narrow and almost as deep as lateral ones, protuberances between median and lateral emarginations wide and nearly parallel-sided; ventral process of each paramere wide.

##### Description.

**Male** (Fig. 8). Head yellow, with a inverse-trapeziform black marking on vertex, clypeus and mouthparts light yellow, apices of mandibles, terminal labial and maxillary palpomeres dark brown, antennae black, pronotum yellow, scutellun light yellow, elytra light yellow, slightly darkened at apices, legs yellow, femora slightly darkened at apices , tibiae black along upper sides, tarsi black, ventral parts of thorax and abdomen black, posterior and lateral margins of each abdominal ventrite and the whole last ventrite yellow.

Head subquadrate, evenly narrowed behind eyes, dorsum densely and finely punctate, eyes slightly protruding, breadth across eyes wider than anterior margin of pronotum, terminal maxillary palpomeres long-triangular, widest near apices, antennae filiform and simple, extending to middle of elytra, antennomeres II about twice as long as wide, III about twice as long as II, V longest, XI slightly longer than X.

Pronotum subquadrate, slightly wider than long, widest at base, anterior margin arcuate, lateral margins diverging posteriorly, posterior margin almost straight, anterior angles rounded, posterior angles nearly vertical, disc densely and finely punctate as that on head, slightly convex on posterolateral parts.

Elytra about 5 times longer than pronotum, 4 times longer than humeral width, lateral margins slightly diverging posteriorly, disc slightly sparsely and largely punctate than that on pronotum.

Legs: all tarsal claws bifid, with lower claws slightly shorter than upper ones.

**Figures 23–31.**
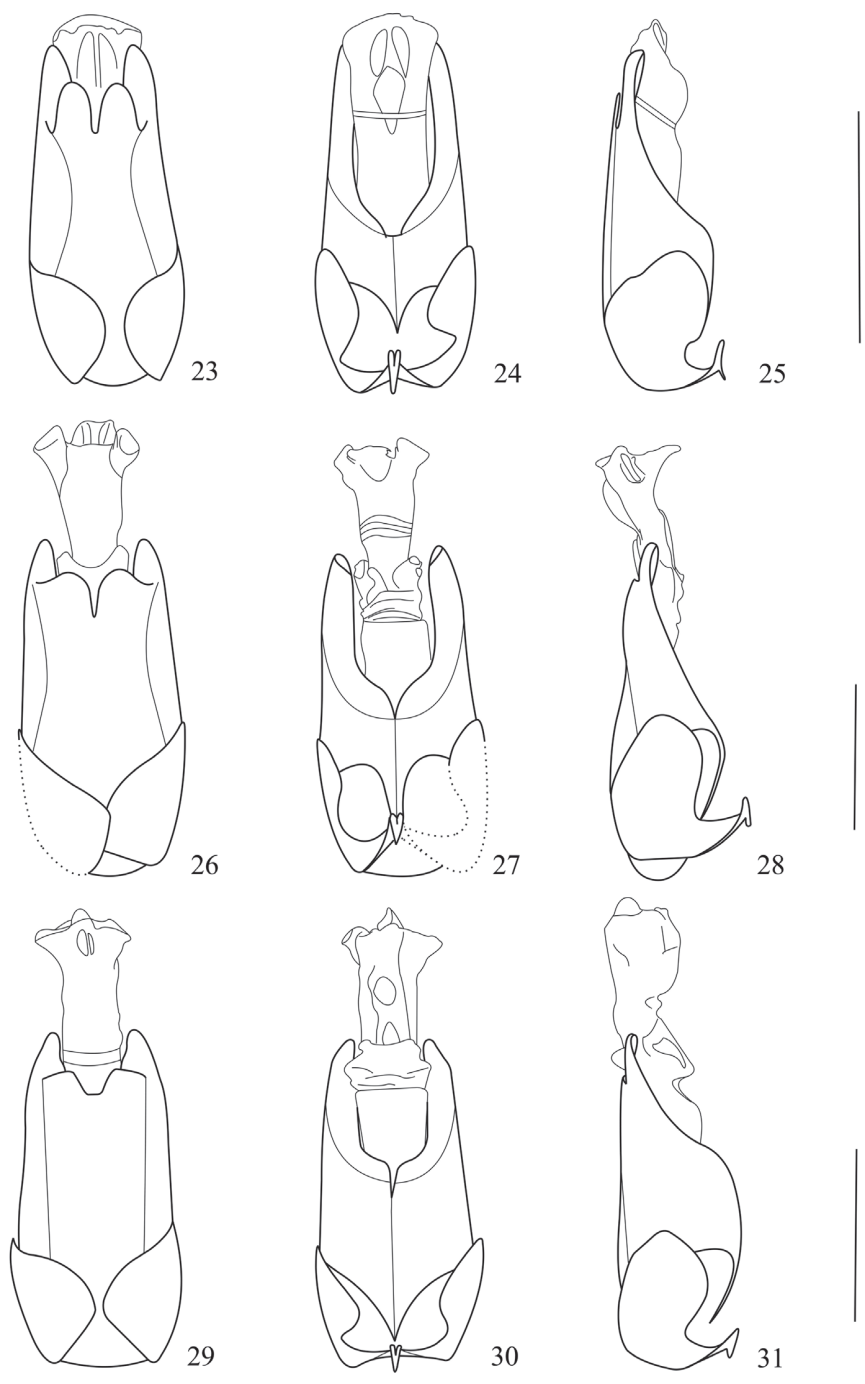
Aedeagi **23–25** *Fissocantharis maculiceps* sp. n. **26–28** *Fissocantharis bimaculata* sp. n. **29–31** *Fissocantharis flava* sp. n. **23, 26, 29** dorsal view **24, 27, 30** ventral view **25, 28, 31** lateral view. Scale bars: 1 mm.

Aedeagus (Figs 23–25): conjoint dorsal plate of parameres with median emargination of apical margin narrow and almost as deep as lateral ones, protuberances between median and lateral emarginations wide and nearly parallel-sided and rounded at apices, about half length of ventral process of each paramere in dorsal view; ventral process of each paramere wide, slightly turned outwards at apex in lateral view; median lobe without any sclerotized projection in dorsum.

**Female.** Body larger, eyes less protruding, antennae shorter and narrower than that of males, pronotum with disc slightly convex, elytra with lateral margins slightly diverging posteriorly, tarsal claws with lower claws distinctly shorter than upper ones.

##### Variation in type series.

Sometimes head with a small rounded black marking on vertex, elytra entirely light yellow, legs with femora and tibiae entirely yellow. Body length: 7.0–9.0 mm; width: 1.6–2.0 mm.

##### Etymology.

This new specific name is derived from Latin *macula* (marking) and *ceps* (head), referring to its head with a black marking on vertex.

#### Fissocantharis bimaculata sp. n.

urn:lsid:zoobank.org:act:23308F99-71D6-40D9-BB4B-3B3F126A8DD8

http://species-id.net/wiki/Fissocantharis_bimaculata

Figs 9 26–28 

##### Type material.

Holotype ♂, CHINA, Sichuan, Mt. Emei, 1600m, 31.v.1979, leg. Jinwen Shang (IZAS). Paratype: 1♂, same locality, 1600–2100m, 24.vi.1955, leg. Le Wu (IZAS) [both transliterated from Chinese labels].

##### Distribution.

China (Sichuan).

##### Diagnosis.

This new species is related to *Fissocantharis semifumatoides* (Švihla, 2005), but distinguishable by the pronotum distinctly wider than long; elytra each with a black marking at apex, lateral margins distinctly diverging posteriorly.

##### Description.

**Male** (Fig. 9). Body yellow, mouthparts dark brown, antennae black, elytra each with a large rounded black marking at apex, femora slightly darkened at apices, tibiae black along upper sides, tarsi black, meso- and metasterna and abdomen black, posterior and lateral margins of each abdominal ventrite and the whole last ventrite yellow.

Head subquadrate, evenly narrowed behind eyes, dorsum densely and finely punctate, eyes moderately protruding, breadth across eyes slightly narrower than anterior margin of pronotum, terminal maxillary palpomeres long-triangular, widest near apices, antennae filiform and simple, extending to apical one-fifth of elytra, antennomeres II slightly widened apically, about twice as long as wide at apices, III about twice as long as II, V longest, XI slightly shorter than X.

Pronotum subquadrate, distinctly wider than long, widest at base, anterior margin arcuate, lateral margins diverging posteriorly, posterior margin almost straight, anterior angles rounded, posterior angles nearly vertical, disc densely and finely punctate as that on head, distinctly convex on posterolateral parts.

Elytra about 5 times longer than pronotum, 2.5 times longer than humeral width, lateral margins distinctly diverging posteriorly, disc slightly sparsely and largely punctate than that on pronotum.

Legs: pro- and mesotarsal claws bifid, with lower claws slightly shorter than upper ones.

Aedeagus (Figs 26–28): conjoint dorsal plate of parameres with median emargination of apical margin narrow and distinctly deeper than lateral ones, protuberances between median and lateral emarginations wide and rounded at apices, almost as long as ventral process of each paramere in dorsal view; ventral process of each paramere wide, slightly turned outwards at apex in lateral view; median lobe without any sclerotized projection in dorsum.

Body length: 9.0–11.0 mm; width: 2.5–3.0 mm.

**Female.** Unknown.

##### Etymology.

This new specific name is derived from Latin *bi-* (two) and *macula* (marking), referring to its elytra each with a black marking at apex.

##### Remarks.

The metalegs of both holotype and paratype and antennomeres III–XI of paratype are missing. Besides, the left basal piece of holotype and basal pieces of aedeagus of paratype are damaged.

#### Fissocantharis flava sp. n.

urn:lsid:zoobank.org:act:CC97E9B5-717A-4CF1-A403-3DFA3F2CFED5

http://species-id.net/wiki/Fissocantharis_flava

Figs 10 29–31 

##### Type material.

Holotype ♂, CHINA, Sichuan, Wanxian, Wangerbao, 1200m, 28.v.1994, leg. Wenzhu Li [transliterated from Chinese label] (IZAS). Paratypes: 1♀, same locality, 27.v.1994, leg. Xingke Yang (IZAS); 1♂, W. Guizhou prov., Leigongshan, Xijiang, 1200–1900m, 29.v–2.vi.1997, lgt. Bolm (IZAS).

##### Distribution.

China (Sichuan, Guizhou).

##### Diagnosis.

This new species is similar to *Fissocantharis bimaculata* sp. n., but differs in the following characters: pronotum slightly wider than long in male; elytra entirely yellow; aedeagus: conjoint dorsal plate of parameres with median emargination of apical margin slightly wide and inverse-trapeziform, protuberances between median and lateral emarginations truncated at apices.

##### Description.

**Male** (Fig. 10). Body yellow, apices of mandibles dark brown, antennae black, antennomeres I yellow, slightly darkened at apices, femora black at apices, tibiae black along upper sides, tarsi black, metasternum and abdomen black, posterior and lateral margins of each abdominal ventrite and the whole last 2 ventrites yellow.

Head subquadrate, evenly narrowed behind eyes, dorsum densely and finely punctate, eyes moderately protruding, breadth across eyes slightly wider than anterior margin of pronotum, terminal maxillary palpomeres long-triangular, widest near apices, antennae filiform and simple, extending to apical one-fourth of elytra, antennomeres II slightly widened apically, about twice as long as wide at apices, III about twice as long as II, V longest, XI slightly longer than X.

Pronotum subquadrate, slightly wider than long, widest at base, anterior margin arcuate, lateral margins diverging posteriorly, posterior margin almost straight, anterior angles rounded, posterior angles nearly vertical, disc densely and finely punctate as that on head, distinctly convex on posterolateral parts.

Elytra about 5 times longer than pronotum, 2.5 times longer than humeral width, lateral margins distinctly diverging posteriorly, disc slightly sparsely and largely punctate than that on pronotum.

Legs: all tarsal claws bifid, with lower claws slightly shorter than upper ones.

Aedeagus (Figs 29–31): conjoint dorsal plate of parameres with median emargination of apical margin slightly wide and inverse-trapeziform, distinctly deeper than lateral ones, protuberances between median and lateral emarginations wide and truncated at apices, almost as long as ventral process of each paramere in dorsal view; ventral process of each paramere wide, slightly turned outwards at apex in lateral view; median lobe without any sclerotized projection in dorsum.

**Female.** Body larger, eyes less protruding than that of males, pronotum distinctly wider than long, disc slightly convex, tarsal claws with lower claws distinctly shorter than upper ones.

Body length: 10.0–14.0 mm; width: 2.0–3.5 mm.

##### Etymology.

This new specific name is derived from Latin *flavus* (yellow), referring to its yellow elytra.

##### Remarks.

The female paratype with right antenna, left antennomeres III–XI, left metatarsomere V and right metatarsomeres III–V are missing.

## Supplementary Material

XML Treatment for Fissocantharis semifumata

XML Treatment for Fissocantharis fissa

XML Treatment for Fissocantharis yui

XML Treatment for Fissocantharis semimetallica

XML Treatment for Fissocantharis bicolorata

XML Treatment for Fissocantharis maculiceps

XML Treatment for Fissocantharis bimaculata

XML Treatment for Fissocantharis flava
